# The Etiology of Metabolic Disturbances in Schizophrenia: Drugs, Genes, and Environment

**DOI:** 10.1093/ijnp/pyab047

**Published:** 2021-07-12

**Authors:** Gavin P Reynolds

**Affiliations:** Biomolecular Sciences Research Centre, Sheffield Hallam University, Howard Street, Sheffield, UK

Mizuki et al. (2021) have provided an extensive analysis of the relationship between type 2 diabetes (T2D) and schizophrenia in which they have focused on a possible commonality of genetic risk as well as reviewing in depth some molecular mechanisms that may underlie the relationship between these 2 diseases. There is much to elucidate in this important yet complex topic, which, as these authors acknowledge, remains far from fully understood. Some of the points they raise do, however, deserve further debate. Although this discussion could be more extensive, I wish to address just 3 questions that emerge from this interesting article.

How strong is the link between schizophrenia and T2D? It is well established that there is an elevated incidence of diabetes in people with schizophrenia. The extent of this increased incidence depends strongly on the particular drugs being taken (Koro et al., 2002), indicating that antipsychotic medication, particularly with drugs that also induce weight gain, makes a substantial contribution to this relationship. Other consequences or correlates of schizophrenia, such as higher prevalence of smoking, less exercise, and poor diet, may also contribute to T2D risk, leading to the suggestion from Mizuki et al. (2021) that “schizophrenia itself is a risk for increased onset of diabetes.” More difficult is determining whether there is an inherent association between schizophrenia and T2D independent of the consequences of psychotic symptoms and their treatment. The main approach is to search for T2D risk factors in early-stage, drug-naïve schizophrenia patients.

Unsurprisingly, there is really no evidence for an increased prevalence of T2D in such patients, given that it typically takes many years to develop. Several groups fail to identify any significant metabolic abnormalities at this early stage of psychotic illness, although some reports cited by Mizuki et al. (2021), including the meta-analysis of Pillinger et al. (2017), have identified increases in markers of glucose intolerance and insulin resistance. However, these findings still question whether the effect is truly trait, rather than state, related. For example, a study included in Pillinger et al. (2017) and showing an unusually large elevation in fasting glucose is that of Zhang et al. (2015); their participants had a reported duration of untreated illness of 24 months, during which time the schizophrenia-related behaviors mentioned above may well have contributed to the development of metabolic disturbances. Incidentally, the findings of Zhang et al. (2015) contrast profoundly with an earlier report from the same group in which no elevation compared with controls was found (Chen et al., 2013); this inconsistency reflects a substantial difference in fasting glucose not between the patient groups but between the controls! While Mizuki et al. (2021) identify other evidence for subtle differences in indicators of relative glucose intolerance or insulin resistance in first episode patients, it is important to bear in mind that these measures are not inevitable precursors of T2D.

Mizuki et al. (2021) hypothesize that common genetic factors may underlie the co-existence of T2D and schizophrenia. How strong is the evidence for genetic commonality? These authors identified several candidate genes that overlap between the 2 disorders. However, given the fact that most case-control candidate gene studies are not replicated by genome-wide association studies or of value in identifying genuine associations with disease, as has been demonstrated for schizophrenia (Johnson et al., 2017), it is perhaps unwise to use candidate gene findings to search for genetic commonalities. Perhaps the most useful study addressing genetic overlap between schizophrenia and T2D is in the very recent report from Perry et al. (2021) using genome-wide association studies data from very large cohorts of people with schizophrenia or various cardiometabolic traits. These authors show clearly that there is no genetic commonality between schizophrenia and T2D. However, after restricting their analysis to inflammation-associated markers, they found that genetic factors for insulin resistance (but not T2D) were also associated with schizophrenia, suggesting that inflammation was an etiological factor common to both schizophrenia and insulin resistance. This finding is consistent with one of the hypothesized mechanisms discussed by Mizuki et al. (2021).

Are there other factors that may contribute to the relationship between schizophrenia and T2D? In addition to the various behavioral risk factors for diabetes seen in people with schizophrenia, there are several environmental etiological risks common to both schizophrenia and T2D and that have not been considered by Mizuki et al. (2021). We have previously pointed out how social and economic deprivation, urbanization, migrant background, and poor parental care and childhood trauma are risk factors for both disorders (Reynolds and McGowan, 2017). A potential feature of all these risks is chronic stress; this is associated with dysfunction of the hypothalamic-pituitary-adrenal axis, which can result in, and be further affected by, inflammation (Russell et al., 2018).

There has been little attempt to explore the complex relationships between schizophrenia, metabolic disturbances, and these environmental risk factors. However, 1 environmental factor has been investigated in terms of the overlap between schizophrenia and metabolic dysfunction. The experience of childhood trauma in people with first-episode schizophrenia is associated with increased insulin (and the related c-peptide) (Tosato et al., 2020), providing a possible indication of insulin resistance. Thus, for some people with schizophrenia, their disease may derive from early environmental risk interacting with genetic factors associated with inflammation, a process that may also result in metabolic dysfunction. Of course, as Mizuki et al. (2021) emphasize, schizophrenia is a heterogeneous disorder in both etiology and phenomenology, and this describes only 1 of multiple mechanisms. Finally, we must not let this focus on the inherent relationship between schizophrenia and metabolic disturbances distract us from the severe effects on body weight and consequent metabolic syndrome that can result from several of the drugs used for the treatment of psychosis.

## Statement of Interest

GPR has received honoraria for lectures from Kang Hong, Lundbeck, Otsuka, and Sumitomo.
